# Intratumoral Heterogeneity of MicroRNA Expression in Rectal Cancer

**DOI:** 10.1371/journal.pone.0156919

**Published:** 2016-06-03

**Authors:** Anne Haahr Mellergaard Eriksen, Rikke Fredslund Andersen, Boye Schnack Nielsen, Flemming Brandt Sørensen, Ane Lindegaard Appelt, Anders Jakobsen, Torben Frøstrup Hansen

**Affiliations:** 1 Danish Colorectal Cancer Center South, Vejle Hospital, Vejle, Denmark; 2 Institute of Regional Health Research, University of Southern Denmark, Odense, Denmark; 3 Bioneer A/S, Hørsholm, Denmark; 4 Department of Oncology, Rigshospitalet, University of Copenhagen, Copenhagen, Denmark; UCSF / VA Medical Center, UNITED STATES

## Abstract

**Introduction:**

An increasing number of studies have investigated microRNAs (miRNAs) as potential markers of diagnosis, treatment and prognosis. So far, agreement between studies has been minimal, which may in part be explained by intratumoral heterogeneity of miRNA expression. The aim of the present study was to assess the heterogeneity of a panel of selected miRNAs in rectal cancer, using two different technical approaches.

**Materials and Methods:**

The expression of the investigated miRNAs was analysed by real-time quantitative polymerase chain reaction (RT-qPCR) and *in situ* hybridization (ISH) in tumour specimens from 27 patients with T3-4 rectal cancer. From each tumour, tissue from three different luminal localisations was examined. Inter- and intra-patient variability was assessed by calculating intraclass correlation coefficients (ICCs). Correlations between RT-qPCR and ISH were evaluated using Spearman’s correlation.

**Results:**

ICC_single_ (one sample from each patient) was higher than 50% for miRNA-21 and miRNA-31. For miRNA-125b, miRNA-145, and miRNA-630, ICC_single_ was lower than 50%. The ICC_mean_ (mean of three samples from each patient) was higher than 50% for miRNA-21(RT-qPCR and ISH), miRNA-125b (RT-qPCR and ISH), miRNA-145 (ISH), miRNA-630 (RT-qPCR), and miRNA-31 (RT-qPCR). For miRNA-145 (RT-qPCR) and miRNA-630 (ISH), ICC_mean_ was lower than 50%. Spearman correlation coefficients, comparing results obtained by RT-qPCR and ISH, respectively, ranged from 0.084 to 0.325 for the mean value from each patient, and from -0.085 to 0.515 in the section including the deepest part of the tumour.

**Conclusion:**

Intratumoral heterogeneity may influence the measurement of miRNA expression and consequently the number of samples needed for representative estimates. Our findings with two different methods suggest that one sample is sufficient for adequate assessment of miRNA-21 and miRNA-31, whereas more samples would improve the assessment of miRNA-125b, miRNA-145, and miRNA-630. Interestingly, we found a poor correlation between the expression estimates obtained by RT-qPCR and ISH, respectively.

## Introduction

MicroRNAs (miRNAs) are short non-coding RNA molecules acting as negative gene regulators at the post-transcriptional level. It is well known that the same miRNA may affect several genes, but also that the same gene may be downregulated by different miRNAs. Thus, miRNAs represent a complex regulatory system of central biological importance. The system is dysregulated during malignant transformation, and it is generally anticipated that change of miRNA activity plays a central role in carcinogenesis [1–4]. Consequently, a considerable and increasing number of studies have investigated miRNAs as potential markers of diagnosis, treatment, and prognosis. The clinical implications, however, have so far been minimal [5]. One reason may be different methodological approaches, which hampers comparison of studies. Another issue is the different composition and quality of patient materials investigated. Moreover, the literature is dominated by many small studies without subsequent validation of relevant findings [5–10].

Another important obstacle is normalisation of real-time quantitative polymerase chain reaction (RT-qPCR) data, when measuring miRNA expression, as appropriate normalisation is important to ensure reliable and reproducible results. Despite an increasing number of miRNA expression studies, the literature is sparse when it comes to reliable normaliser candidates [11–14].

Intratumoral heterogeneity is a general characteristic of malignant tumours [15–17] and represents a major obstacle in the study of tumour biology, miRNAs being no exception. To date, the issue has been almost neglected despite its obvious importance; the activities of miRNAs are likely to vary in different parts of the tumour with potential major impact on the clinical application. Only few publications have addressed the problem of intratumoral heterogeneity affecting the measurement of miRNA expression. One study is dealing with the issue in breast cancer [18], another one in hepatocellular carcinoma [19].

These challenges are especially relevant in patients with locally advanced rectal cancer, where treatment decisions are often based on single biopsies and the later interpretation of the tumour is influenced by the pre-operative radiotherapy [20, 21].

The aim of the present study was to analyse the expression heterogeneity of a panel of selected miRNAs in rectal cancer specimens by two different technical approaches, and to elucidate their mutual concordance. RT-qPCR was chosen, since it is widely accepted as the gold standard for miRNA expression analyses. *In situ* hybridization (ISH) was chosen because of its ability to visualise the cellular localisation of the investigated miRNAs in addition to the expression level.

## Materials and Methods

### Patients and tissue samples

Twenty-seven patients were randomly selected from a cohort of 239 patients who had undergone resection for rectal cancer at Vejle Hospital, Denmark from 1999 to 2008. Inclusion was made only if the histopathological diagnosis was rectal adenocarcinoma pT3 or pT4, if no preoperative chemo-radiotherapy was given, and if formalin-fixed paraffin-embedded (FFPE) rectal cancer tissue was available from three different localisations of the tumour, including the luminal aspect.

According to The Regional Scientific Ethical Committees for Southern Denmark, ethics approval and written informed consent were not required, since the purpose of the study was to develop new methodology and no clinical data were further analysed (Act on Research Ethics Review of Health Research Projects, cf. § 14, section 1). The samples were collected during standard rectal resection, and the project was carried out without knowledge of the clinical data. The study was registered with The Danish Data Protection Agency, and The Danish Registry of Human Tissue Utilisation was consulted before any tissue samples were used.

Available histological sections stained with hematoxylin and eosin (H&E) were examined by a pathologist in order to select three localisations from each tumour. The sections had to be cut from tissue blocks sampled from three different localisations, and all had to include the luminal aspects of the tumour mirroring the locations where diagnostic, endoscopic biopsies would have been taken. According to this selection, adjacent sections were cut from each of the FFPE tissue blocks for the heterogeneity analyses as follows: (a) one section to be stained with H&E for identification of Region of Interest (ROI), (b) four sections for ISH (5 μm), (c) two sections to be stained with hematoxylin for RT-qPCR (8 μm), (d) one look-up section to be stained with H&E. On a print of the H&E stained section, tumour and tumour microenvironment were identified by the pathologist and encircled as regions of interest (ROI). Subsequently, the marking was transferred to the digital whole slide image of the ISH-slide, and to the H&E stained section and the hematoxylin stained section for RT-qPCR. For the RT-qPCR-analysis, the marked area was removed using a scalpel and collected in 1.5 ml RNase-free PCR tubes containing a drop of ethanol (99%) (S1 and S2 Figs).

### Target miRNAs and normaliser miRNAs

Five target miRNAs were chosen based on the literature searches: miRNA-21 based on its well-known up-regulation in colorectal cancer (CRC) and suggested down-regulation after neo-adjuvant chemo-radiotherapy [6, 9, 22]; miRNA-31 based on the positive correlation between expression level and stage of CRC [4, 23]; miRNA-125b, miRNA-145, and miRNA-630 based on their suggested up-regulation in rectal cancer tissue after neo-adjuvant chemo-radiotherapy [6–8].

Addressing the question of normalisation we recently performed a study on miRNA expression profiling to identify and validate reference genes for relative quantification of miRNAs. MicroRNA-193a-5p, miRNA-27a, and let-7g were identified as the most stably expressed miRNAs in our cohort of rectal cancer and were consequently used as normalisers in the present study [24].

### Expression analysis by RT-qPCR

#### RNA extraction

RNA was isolated from the micro dissected FFPE tissue using the miRNeasy FFPE Kit (Qiagen, Germany) according to the manufacturer’s instructions. First step was adding a lysis buffer containing proteinase K followed by a short incubation at 70°C. This was followed by DNase treatment and RBC buffer treatment. Ethanol was added and the sample was applied to an RNeasy MinElute spin column, where the total RNA, including miRNA, was bound to the membrane. Finally, total RNA was eluted in 14 μl RNase-free water.

#### RT-qPCR

The Custom TaqMan® MicroRNA Single Assays (Life Technologies, CA, USA) for hsa-let-7g 002282, hsa-miR-193a-5p 002281, hsa-miR-27a 000408, hsa-miR-21 000397, hsa-miR-31 002279, hsa-miR-125b 000449, hsa-miR-145 002278, and hsa-miR-630 001563 were used according to the manufacturer’s standard instructions for low sample input (LSI).

Custom MicroRNA RT Primer Pool and Custom MicroRNA PreAmp Primer Pool for analysing the above mentioned miRNAs were created according to Protocol for Creating Custom RT and Preamplification Pools using TaqMan® MicroRNA Assays (Publication Part Number 4465407, Life Technologies).

RNA was reverse-transcribed using the Custom MicroRNA RT Primer Pool. Each RT reaction contained 3 μl total RNA and 12 μl RT reaction mix from TaqMan® MicroRNA Reverse Transcription Kit.

Preamplification (12 cycles) was performed with the Custom MicroRNA PreAmp Primer Pool, each reaction containing 2.5 μl RT Product, TaqMan® PreAmp Master Mix (2X) and PreAmp Primer Pool in a total reaction volume of 25 μl. The PreAmp Product was diluted 1:40 in TE buffer.

The RT-qPCR analyses were carried out on Applied Biosystems 7900 HT Real-Time System using 1 μl diluted PreAmp product, TaqMan® MicroRNA Assays and TaqMan® Universal Master Mix II NoAmpErase® UNG in a total reaction volume of 20 μl. All reactions were performed in triplicate. Data analysis was performed using SDS software ver. 2.2.2 (Life Technologies).

Water was used as negative control. The no template control was included in the entire process (RT, preamplification and qPCR) and analysed together with samples.

#### Relative quantification

Average values of triplicate Cq values were used for further analysis. Cq is defined as the PCR cycle number at which the fluorescence meets the threshold in the amplification plot. The arithmetic mean of Cq values for miRNA-193a-5p, miRNA-27a, and let-7g was used for normalisation as mentioned above.

ΔCq values were converted to linear values for statistical analyses by the equation linear value (ΔCq) = *2*^*-ΔCq*^ assuming that the amplification efficiency of the assays was close to 100%.

### Expression analysis by in situ hybridization

Prior to the analysis, assay optimisation was accomplished to determine optimal probe concentrations and hybridization temperatures. MicroRNA-31 was not detected in 10 samples analysed during assay optimisation and was therefore not further explored. ISH for miRNA-21, miRNA-125b, miRNA-145, and miRNA-630 was performed as previously described [25] using the following concentrations: 20nM (miRNA-21 and miRNA-145), 30nM (miRNA-630), 50nM (miRNA-125b). The ISH analysis was performed using a Tecan Freedom Evo automated hybridization instrument (Tecan, Switzerland), in which the following steps were performed: pre-digestion with proteinase-K (15 μg/ml) at 37°C for 8 minutes, pre-hybridization at 57°C for 15 minutes, hybridization with double-carboxyfluorescein (FAM) labelled Locked Nucleic Acid (LNA) probes (Exiqon A/S, Denmark) for 1 hour. The miRNA-125b was processed at 56°C. After stringent washes with saline-sodium-citrate buffer the probes were detected with alkaline phosphatase-conjugated sheep anti-FAM Fab fragments, followed by incubation in 4-nitroblue tetrazolium and 5-bromo-4-chloro-3’-indolylphosphate (Roche, Denmark) for 60 minutes (90 minutes for miRNA-125b) resulting in a dark blue staining, and finally counterstaining with nuclear fast red (Vector Laboratories, CA, USA).

### Image analysis and quantification

Digital whole slide images were obtained with an x20 objective using an Axio Scan Z1 bright field scanner (Carl Zeiss, Germany). Image analysis of the digital slides was performed using VisiomorphDP software (Visiopharm, Denmark).

The areas of ROI encircled on the H&E stained sections were outlined on the ISH digital whole slide images, and staining artefacts as well as tissue artefacts were cleared. Some slides were in a poor condition caused by folded or lost tissue, which limited the size of the area included in the ROI. We found, not surprisingly, that images with a small ROI (less than 2 mm^2^) contributed with quite dramatic variation and they were therefore omitted from the statistical analyses. For miRNA-21 five patients were excluded; for miRNA-125b and miRNA-630 three patients were excluded; and for miRNA-145 four patients were excluded.

A pixel classifier was trained to discriminate the blue ISH signal from the red counterstain, the unstained and weakly stained tissue, and the tissue-free areas. The following parameters were obtained during image processing from each ROI: area of intense blue (the ISH signal), area of weak blue (considered background ISH signal), area of purple blue (blue located over nuclear red), and the total area of the individual ROIs. The relative area fractions (areas of blue colour of interest divided by the total ROI area) were considered representative of the relative expression of the miRNA of interest.

### Data analysis

All data were summarised using standard descriptive statistics. Inter- and intra-patient variability was assessed by calculation of intraclass correlation coefficients (ICC), using a one-way random-effects model. Specifically, the ICC may in this setting be regarded as the percentage of the total variance in the samples accounted for by differences between the patients examined. If the majority of variation in the sample measurements is from inter-patient variation, the ICC is high (ICC → 1). If the majority of variation is from intra-patient variation, the ICC is low (ICC → 0). We considered ICC both for single samples (ICC_single_) and for the mean of samples within patients (ICC_mean_). The ICC_single_ provides an estimate of the reliability of a given sample as to rendering information about the specific patient (compared to all other patients). Correspondingly, the ICC_mean_ estimates the reliability of the information rendered by taking the mean of three samples from a patient.

The correlation between RT-qPCR and ISH-fractions for each miRNA was estimated for individual samples and for mean values for individual patients using the Spearman correlation coefficient.

In order to illustrate the inter- and intra-patient variation as well as the correlation between RT-qPCR and ISH values, all data were normalised to allow for plotting on a common scale. For each miRNA, the mean and the standard deviation for all RT-qPCR measurements of each patient were calculated, and any individual sample measurement was normalised to a standard scale by extracting the mean and dividing it by the standard deviation. This was also done for all ISH measurements, for each miRNA. The resulting values for both RT-qPCR and ISH were plotted separately for each miRNA.

## Results

### RT-qPCR

The expression pattern of the target miRNAs (miRNA-21, miRNA-31, miRNA-125b, miRNA-145, and miRNA-630) was analysed in three different samples from each tumour specimen by RT-qPCR. MicroRNA-21, miRNA-31, miRNA-125b, and miRNA-145 showed different and measureable expression levels, and all of the miRNAs were expressed in all three samples from each tumour. MicroRNA-630 was undetected in two samples from two different patients. The range of raw Cq levels was 17.4–21.6 for miRNA-21, 20.9–28.7 for miRNA-31, 20.4–26.3 for miRNA-125b, 15.9–24.4 for miRNA-145, and 28.5–36.7 for miRNA-630. A few outliers of the triplicate RT-qPCR measurements were removed from the dataset before further analyses. After normalisation, the ΔCq values were converted to linear values for further analysis.

### In situ hybridization

**Fig 1:**
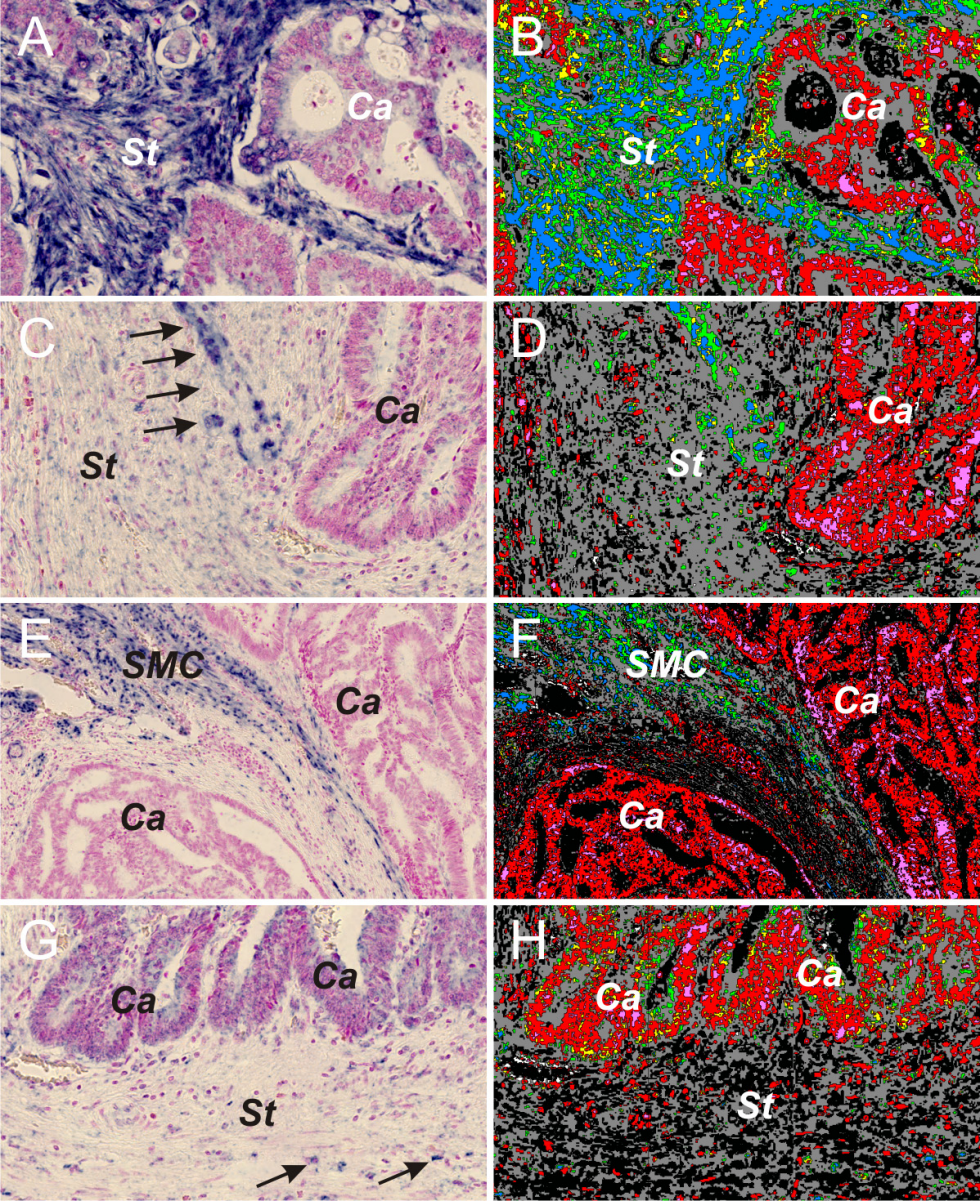
*In situ* hybridization (ISH) visualizing the expression of miRNA-21, miRNA-125b, miRNA-145, and miRNA-630 in rectal cancer tissue. The ISH signal is corresponding to the blue colour in the left row. St: stroma; Ca: epithelial tumor cells. **(A)** Expression of miRNA-21 mainly present in the stroma adjacent to the epithelial tumour cells. **(B)** Pixel classifier corresponding to image A. **(C)** Expression of miRNA-125b with strong ISH-signal present in an enteric neuron (arrows) and weaker ISH signal representing different stromal cells. **(D)** Pixel classifier corresponding to image C. **(E)** Expression of miRNA-145 mainly present in smooth muscle cells (SMC) and myofibroblastic cells. **(F)** Pixel classifier corresponding to image E. **(G)** Expression of miRNA-630 scattered in epithelial tumour cells and adjacent stromal cells (arrows indicate examples of ISH-positive cells in the stroma). **(H)** Pixel classifier corresponding to image G.

Relative expression estimates for the target miRNAs (miRNA-21, miRNA-125b, miRNA-145, and miRNA-630) were obtained by image analysis of the ISH-stained slides (Table 1).The miRNA-21 ISH resulted in a strong signal in the stromal tissue surrounding the epithelial tumour cells as also described previously [25, 26]. A strong signal in enteric neurons in the stromal tissue and a weaker signal in other stromal cells were detected for miRNA-125b. The ISH for miRNA-145 resulted in a strong and a weak signal mainly in the smooth muscle cells and myofibroblastic cells surrounding the tumour cells [26, 27]. The miRNA-630 ISH showed staining of tumour cells and a subset of the adjacent stromal cells. Both strong and weak ISH signals were considered in the image analysis. Examples of the ISH signals for miRNA-21, miRNA-125b, miRNA-145, and miRNA-630 with the corresponding classified images are shown in Fig 1.

**Table 1:** *In situ* hybridization (ISH) for miRNA-21, miRNA-125b, miRNA-145, and miRNA-630: Classifiers, relative area fractions and localisation of ISH signal.

	Classifier (ISH signal)	Area fraction	Localisation of ISH signal
**miRNA-21**	TBp*	0.0091–0.5751	Stromal cells
**miRNA-125b**	TBp	0.0004–0.0848	Stromal cells
	iB**	0.0000–0.0030	Enteric neurons in stroma
	wpB***	0.0004–0.0818	Other stromal cells
**miRNA-145**	TBp	0.0002–0.1141	Smooth muscle cells
**miRNA-630**	TBp	0.0012–0.1302	Tumour cells and stromal cells

Intense blue (iB) represents the strong ISH signal, weak blue (wB) the background ISH signal, and purple blue (pB) the blue located over nuclear red.

* TBp (all blue colours) = iB + wB + pB

** For miRNA-125b, iB represents enteric neurons in the stromal tissue. They were present regardless of cancer and were excluded from the ICC analysis.

*** wpB = TBp—iB; used for calculating the ICC for miRNA-125b.

### Intraclass correlation coefficient (ICC)

The ICC of each miRNA was calculated for both RT-qPCR and ISH as shown in Table 2. For miRNA-21, miRNA-145, and miRNA-630, the TBp-fraction (i.e. all blue colours) was used, but for miRNA-125b we did not include the areas with intense blue staining representing the enteric neurons.

**Table 2:** Intraclass Correlation Coefficient (ICC) for one sample from each tumour (ICC_single_) and for three different samples from each tumour (ICC_mean_) as obtained by RT-qPCR and ISH.

	Method	ICC (single)	ICC (mean)
**miRNA-21**	PCR	**0.650**	**0.848**
	ISH	**0.632**	**0.838**
**miRNA-125b**	PCR	0.453	**0.713**
	ISH	0.281	**0.540**
**miRNA-145**	PCR	0.134	0.317
	ISH	0.264	**0.518**
**miRNA-630**	PCR	0.284	**0.543**
	ISH	0.222	0.461
**miRNA-31**	PCR	**0.782**	**0.915**

The ICC_single_ was higher than 50% for miRNA-21 (RT-qPCR: 65%, ISH: 63%) and miRNA-31 (RT-qPCR: 78%), and for miRNA-125b, miRNA-145, and miRNA-630, it was lower than 50%.

As to ICC_mean_ it was higher than 50% for miRNA-21(RT-qPCR and ISH), miRNA-125b (RT-qPCR and ISH), miRNA-145 (ISH), miRNA-630 (RT-qPCR), and miRNA-31 (RT-qPCR) and lower than 50% for miRNA-145 (RT-qPCR) and miRNA-630 (ISH). As shown in Table 2, the ICC_mean_ is generally higher than the ICC_single_.

### Correlation between qPCR and ISH

The Spearman Correlation Coefficient was calculated to compare the results obtained by RT-qPCR and ISH, respectively (Table 3). For ISH, when comparing with the RT-qPCR, the TBp-fraction (all blue colours) was used for all four miRNAs, since RT-qPCR measures the total expression of the miRNA of interest, regardless of localisation. In general, there was a poor correlation between the estimates obtained by the two techniques with correlation coefficients ranging from 0.084 to 0.325 for the mean value for each patient, and ranging from -0.085 to 0.515 in the section including the deepest part of each tumour.

**Table 3:** Spearman Correlation Coefficients comparing RT-qPCR and ISH for average measures for individual patients and for a single sample from each patient (the one of the three sections including the deepest part of the tumour).

	Spearman Correlation (mean)	Spearman Correlation (deepest section)
**miRNA-21**	0.158	0.294
**miRNA-125b**	0.084	-0.085
**miRNA-145**	0.273	0.312
**miRNA-630**	0.325	0.515

**Fig 2:**
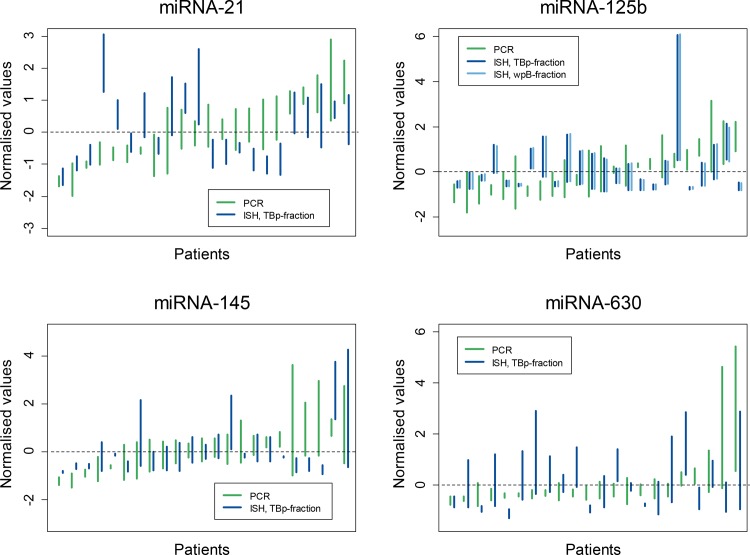
Inter- and intra-patient variation and correlation between RT-qPCR and ISH measurements for miRNA-21, miRNA-125b, miRNA-145, and miRNA-630. All data were normalised to be plotted on a common scale: see the main text for details. Each vertical line represents the range of the three measures obtained in the individual patient with different colours to represent RT-qPCR and ISH measurements, respectively. For each miRNA, patients were sorted according to their mean RT-qPCR measurement for plotting.

The intra- and inter-patient variation and the correlation between RT-qPCR and ISH for miRNA-21, miRNA-125b, miRNA-145, and miRNA-630 are illustrated in Fig 2. For miRNA-31 (RT-qPCR, only) see S3 Fig.

## Discussion

An increasing number of studies focus on miRNAs as potential biomarkers in cancer diagnosis and treatment, but the divergent and contradicting results in the literature are a major obstacle to clinical application [5, 21]. The situation calls for a critical approach to methodological aspects among which heterogeneity ranks high.

Tissue heterogeneity is a general characteristic of malignant tumours. It is well known that the micro-environment varies throughout the tumour with effect on growth, proliferation, and metastatic potential. The behaviour of tumour mutations is inconsistent, too [16, 17]. Consequently, the issue holds crucial importance in biomarker research with perspectives of clinical application. This also applies to miRNAs. The present study addressed the heterogeneity problem comparing two different methods of miRNA expression analysis.

The literature dealing with miRNA and intratumoral heterogeneity is sparse. Raychaudhuri *et al*. (2012) investigated intratumoral heterogeneity of a panel of miRNAs in breast cancer [18] by RT-qPCR. They found a coefficient of variation (CV) of 40% within primary tumour samples and conclude that, intratumoral heterogeneity can lead to significant sampling bias and, assessment of miRNA profiles may require sampling at several different tumour locations. Peveling-Oberhag *et al*. (2014) [19] performed a miRNA profiling in hepatocellular carcinoma comparing different compartments of liver tumours and found that the miRNA dysregulation varies within tumour compartments. The two studies cannot without reservation be compared with our results, as none of them report the ICC. On the other hand, they both report significant intratumoral heterogeneity in accordance with the present study.

To the best of our knowledge, this is the first study to assess the intratumoral heterogeneity of miRNAs in rectal cancer. Moreover, we are not aware of any other studies applying the two techniques of RT-qPCR and ISH on adjacent sections of tissue to elucidate their mutual concordance. RT-qPCR was chosen, since it is widely accepted as the gold standard for miRNA expression analyses. ISH was chosen because of its ability to visualise the cellular localisation of the investigated miRNA in addition to the expression level. We used tumour specimens from patients with rectal cancer to analyse the intratumoral heterogeneity of a panel of miRNAs with potential clinical relevance [4, 6–9, 22, 23].

We found the overall extent of heterogeneity, as expressed by ICC, to be different for the five miRNAs. The CV is not as useful a parameter in this setting, as it does not provide a direct measure of the intra-patient variation relative to the overall variation in the measurements. The ICC, however, allows for comparison of the intra- and inter-patient variation in the datasets from the two different methods, thus providing an estimate of the precision of the individual measurements referring to a specific patient.

The inter-patient variation was higher than that of the intra-patient variation with respect to miRNA-21 and miRNA-31. This is an indication that the two miRNAs have potential as biomarkers, when analysing one sample (e.g. one diagnostic biopsy). Here we found that mean ICC values for RT-qPCR and ISH were almost identical for miRNA-21, 0.848 and 0.838, respectively. These values are in good concordance with Nielsen *et al*. (2011), who reported an ICC value for miRNA-21 ISH of 84% in CRC tissue [25].

On the other hand, for miRNA-125b, miRNA-145, and miRNA-630, the ICC_single_ was below 50%, requiring more cautiousness when interpreting results from a single sample. For miRNA-125b and miRNA-630 the ISH analysis showed lower ICC values (single and mean) than the RT-qPCR analysis, indicating a higher intra-patient variation for the ISH analysis. For miRNA-145 it was an opposite scenario with the ISH analysis showing higher ICC values (single and mean) than the RT-qPCR analysis, indicating a higher intra-patient variation for the RT-qPCR analysis. Therefore, relying only on the ICCs, none of the two techniques are superior to the other.

Interestingly, we found a poor correlation between the results from RT-qPCR and ISH for all the miRNAs; so even though ICC (single and mean) is equal for RT-PCR and ISH for miRNA-21, the results are not comparable. The main explanation is likely to be inherent differences in the methodology of RT-qPCR and ISH. E.g. different from the RT-qPCR technique, the ISH technique detects both precursor and mature miRNAs. However, it cannot be excluded that part of an explanation for the poor correlation could be small displacements of ROI when copying it from the primary plotting to the ISH digital whole slide image and to the slide for RT-qPCR. This would mean a difference in the analysed area, especially in the stroma, where all the miRNAs are represented.

In general, RT-qPCR has a high reproducibility and is a relatively low cost method applicable in most laboratories. FFPE tissue is well suited for miRNA expression analysis performed on a small amount of tissue with only the mature miRNAs being detected. However, RT-qPCR includes several preparatory steps (RNA extraction, reverse transcription, preamplification) in which technical variation can be introduced, and in addition proper normalisation is essential to ensure reproducible results.

The ISH technique presents several advantages, including the ability to visualise the cellular localisation of the investigated miRNAs in addition to the expression level. The quantitative ISH method requires standardised section thickness, because the quantitative estimates are not normalised against a reference standard. Other preparatory steps that may introduce some technical variation include proteolytic pre-treatment as well as tissue folds and detachment. In addition, ISH is a more laborious method not suitable for all laboratories, and data interpretation requires specialised staff

In the present study it was possible to visualise the cellular localisation of the investigated miRNAs, and the corresponding ISH signal allowed for focus on the relevant cells in the ISH data analysis. For miRNA-125b we could leave out the strong ISH signal form the enteric neurons when calculating the ICC. For RT-qPCR, the same option is not available, and miRNA-125b from the enteric neurons is a part of the measured expression level. Since the enteric neurons are present regardless of cancer, leaving them out of the analysis would be the most accurate thing to do. In that case, RT-qPCR including miRNA-125b from the enteric neurons would give a biased estimate, and ISH would probably give a more correct result. This has to be balanced with the loss of cases in the ISH analyses due to folded or lost tissue. Thus, several cases were actually lost for all the analysed miRNAs, probably due to erroneous fixation of the tumours.

For all miRNAs ICC_mean_ was higher than ICC_single_. A low ICC value could be explained as a high measurement noise, whereas a high ICC value indicates less measurement noise. The fact that ICC_mean_ was higher than ICC_single_ suggests that pooling all three samples reduces the measurement noise, but only one available biopsy is often the scenario in clinical practice.

Relying on the ICCs calculated here, it was not possible to conclude which of the two techniques, RT-qPCR or ISH, provides the most representative expression estimates and thereby comprise the tumour heterogeneity. However, for clinical application of a method, practical factors such as number of biopsies needed for examination, accessibility and cost of the method, and interpretation of the results are indeed important factors

In conclusion, intratumoral heterogeneity should be considered in the analysis of miRNAs as biomarkers, and miRNA analysis should hence be performed on all available samples. One sample may be sufficient in some settings as indicated for miRNA-21 and miRNA-31 in the current study. The poor correlation between RT-qPCR and ISH suggests that results obtained by the two techniques should be compared with caution. In a clinical setting, RT-qPCR seems preferable as an easy method of getting reproducible results, however, it cannot be excluded that the ISH method may provide more clinically relevant expression estimates.

## Supporting Information

S1 Fig Rectal cancer specimen for miRNA expression analyses. **(A)** From the superficial / luminal part of the tumour, three different locations (red arrows) were chosen for examination. **(B)** The sections for RT-qPCR and ISH were cut at the same time as adjacent sections. The Region of Interest (ROI) was encircled on a print of the digital whole slide image of the HE-section. ROI was transferred to the digital whole slides of the ISH-images and to the sections cut for RT-qPCR.
(TIF)

S2 Fig Region of Interest (ROI). **(A)** On a print of the H&E stained section, ROI was marked by the pathologist. **(B)** The marking was transferred to the sections stained with both hematoxylin and eosin / stained with hematoxylin only.
(TIF)

S3 Fig Inter- and intra-patient variation for miRNA-31(RT-qPCR only). Data were normalised for plotting on a common scale. For any given measurement, the mean and the standard deviation for that measurement for all samples were calculated, and any individual data point was normalised to a standard scale by extracting the mean and dividing it by the standard deviation. The resulting values for RT-qPCR were plotted for each patient.
(EPS)

S1 Table Data from the RT-qPCR analysis. (XLSX)

S2 Table Data from the ISH image analysis. (XLSX)
