# Supplementary figures and images for: Intratumoral Heterogeneity of MicroRNA Expression in Rectal Cancer
Source: PLoS One. 2016 Jun 3;11(6):e0156919. doi: 10.1371/journal.pone.0156919 (PMC4892647; doi:10.1371/journal.pone.0156919)

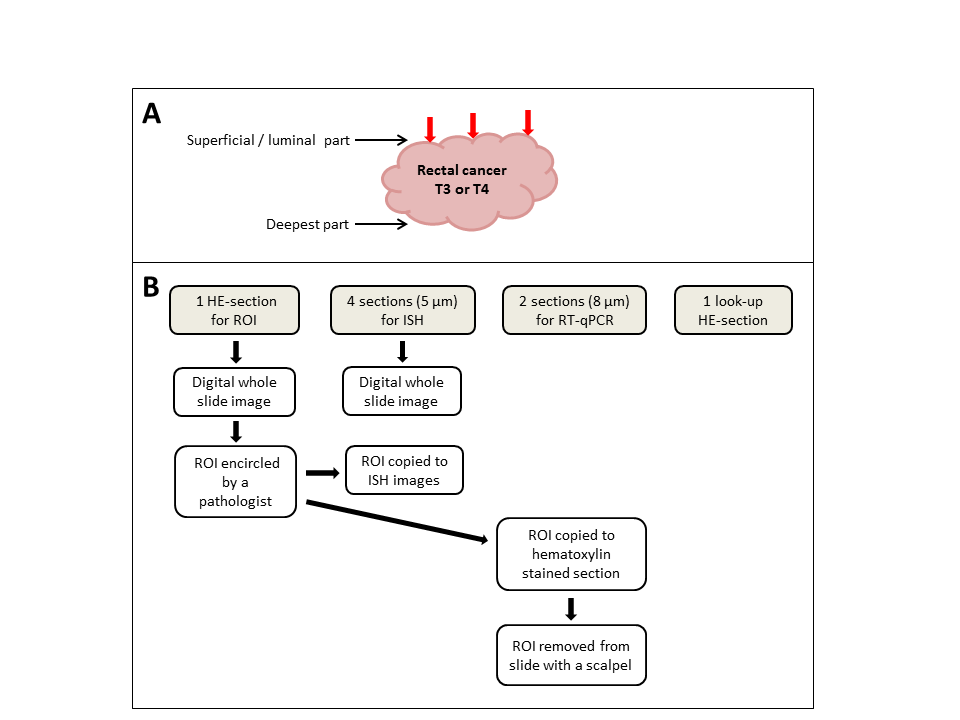

Supplement: S1 Fig — (A) From the superficial / luminal part of the tumour, three different locations (red arrows) were chosen for examination. (B) The sections for RT-qPCR and ISH were cut at the same time as adjacent sections. The Region of Interest (ROI) was encircled on a print of the digital whole slide image of the HE-section. ROI was transferred to the digital whole slides of the ISH-images and to the sections cut for RT-qPCR. (TIF) [file pone.0156919.s001.tif]

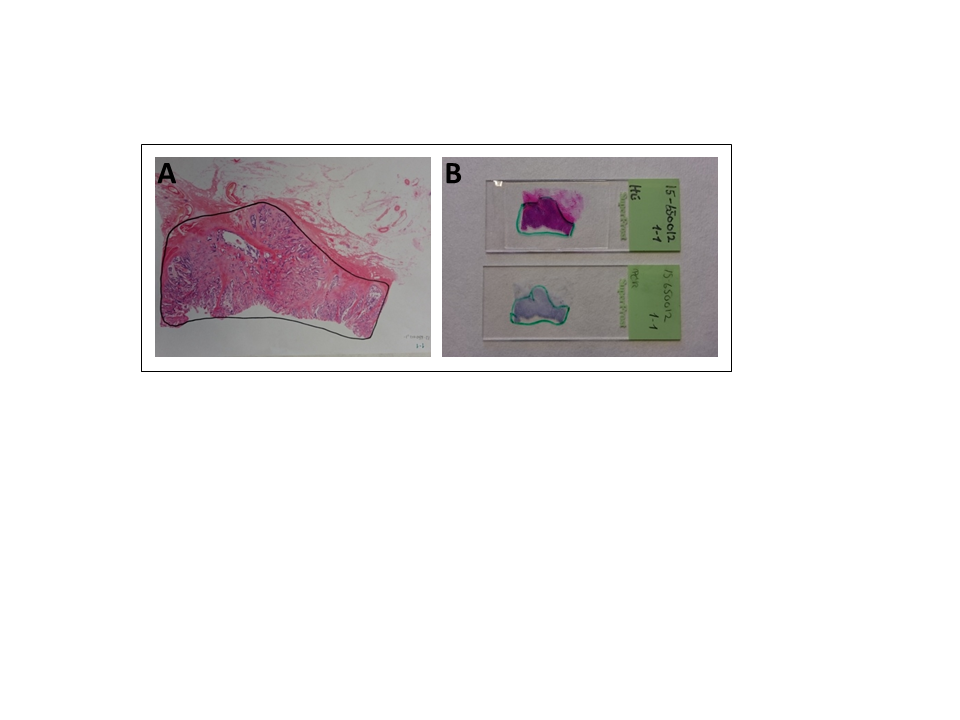

Supplement: S2 Fig — (A) On a print of the H&E stained section, ROI was marked by the pathologist. (B) The marking was transferred to the sections stained with both hematoxylin and eosin / stained with hematoxylin only. (TIF) [file pone.0156919.s002.tif]
